# Cost-effectiveness analysis on COVID-19 surveillance strategy of large-scale sports competition

**DOI:** 10.1186/s40249-022-00955-3

**Published:** 2022-03-18

**Authors:** Xuechun Wang, Yiru Cai, Bo Zhang, Xiangyu Zhang, Lianhao Wang, Xiangyu Yan, Mingchen Zhao, Yuan Zhang, Zhongwei Jia

**Affiliations:** 1 School of Public Health, Peking University, Beijing, 100191 China; 2 School of Mathematical Sciences, Peking University, Beijing, 100871 China; 3 Center for Statistical Sciences, Peking University, Beijing, China; 4 Center for Intelligent Public Health, Institute for Artificial Intelligence, Peking University, Beijing, China; 5 Peking University Clinical Research Institute, Peking University, Beijing, China

**Keywords:** Cost-effectiveness, Sports competition, Surveillance, COVID-19, Nucleic acid test, Stochastic dynamic model

## Abstract

**Background:**

Nucleic acid test (NAT) could effectively control the spread of COVID-19 caused by large-scale sports competitions. However, quantitative analysis on the appropriate frequency of NAT is scarce, and the cost-effectiveness and necessity of high-frequency NAT remain to be fully explored and validated. This study aims to optimize the COVID-19 surveillance strategies through cost-effectiveness analysis for the Tokyo 2020 Olympic Games and the upcoming Beijing 2022 Olympic Winter Games.

**Methods:**

A total of 18 scenarios were designed regarding the NAT frequency, symptom monitoring, and strengthening close-contact control. An agent-based stochastic dynamic model was used to compare the cost-effectiveness of different NAT scenarios and optimize the surveillance strategies. The dynamics of the proposed model included the arrival and departure of agents, transmission of the disease according to Poisson processes, and quarantine of agents based on regular NATs and symptom onset. Accumulative infections, cost, and incremental cost-effectiveness ratio (ICER) were simulated in the frame of the model. ICER was used to compare the cost-effectiveness of different scenarios. Univariate sensitivity analysis was performed to test the robustness of the results.

**Results:**

In Scenario 16, where the competition-related personnel (CRP) received NAT daily and national sports delegation (NSD) with quarantined infections accepted an additional NAT daily, accumulative infection was 320.90 (90 initial infections), the total cost was (United States Dollar) USD 8 920 000, and the cost of detecting out each infection was USD 27 800. Scenario 16 would reduce the total cost by USD 22 570 000 (avoid 569.61 infections), USD 1 420 000 (avoid 47.2 infections) compared with Scenario 10 (weekly NAT, strengthened close contact control) and Scenario 7 (daily NAT, no strengthened close contact control), respectively. Sensitivity analysis showed that the result was most sensitive to the change in basic reproductive number.

**Conclusions:**

High-frequency NATs such as bidaily, daily, and twice a day were cost-effective. NAT daily for CRP with strengthening close-contact control could be prioritized in defense against COVID-19 at large-scale sports competitions. This study could assist policymakers by assessing the cost-effectiveness of NAT scenarios and provide the host country with an optimal COVID-19 surveillance strategy.

**Graphical Abstract:**

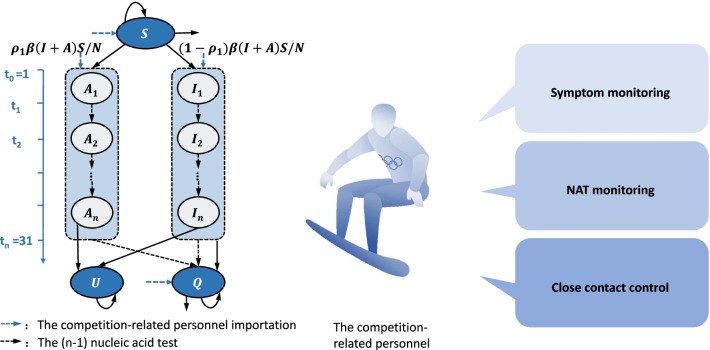

**Supplementary Information:**

The online version contains supplementary material available at 10.1186/s40249-022-00955-3.

## Background

Large-scale sports competitions [1] are defined as various intercontinental and worldwide comprehensive or individual competitions held by the world’s sports organizations, such as the Asian Games, the Tokyo 2020 Olympic Games, and the upcoming Beijing 2022 Olympic Winter Games. The most prominent characteristics of large-scale sports competitions are mass gathering and mass mobility [2, 3], which contribute to the trans-regional transmission of the virus. For example, as of September 5, 2021, the Tokyo 2020 Olympic Games recorded a total of 848 confirmed cases of Olympics-related personnel, including 41 athletes [4].

Nucleic acid test (NAT) is a primary means to control the spread of the epidemic, and it is the gold standard [5, 6] for the timely detection and judgment of COVID-19 cases. For example, the US National Basketball Association required regular NAT for unvaccinated athletes [7]; during the Tokyo 2020 Olympic Games, athletes were tested for NAT daily to monitor the spread of COVID-19 [8]. Countries that have employed mass NAT, especially high-frequency NAT [9], believe that it is an essential strategy to control COVID-19 and detect virus carriers in a timely manner. However, there is no unified standardized surveillance policy or measure globally, and the frequency of NAT is based on previous medical experience [10]. Furthermore, the optimization of NAT frequency, symptom monitoring, and close-contact control have not been fully demonstrated under fixed cost. Moreover, the cost-effectiveness and necessity of high-frequency NAT remain to be fully explored and validated.

Deterministic models [11] and stochastic epidemic models [12, 13] are commonly used to describe the spread of infectious diseases. Compared with deterministic models, stochastic models are more suitable for real-life data fitting [14]. Stochastic models often use agent-based dynamic models under the scheme of a continuous-time Markov process [15, 16] to describe the evolution of epidemics. Therefore, this study used an agent-based dynamic model to optimize the surveillance strategy.

A reasonable COVID-19 surveillance strategy, especially the NAT frequency, sets a solid foundation for a safe, successful, and exciting competition. At the same time, this study also provided a pre-warning COVID-19 model during large-scale sports competitions, which could help various countries control the epidemic, save costs, and reduce the impact on athletes' careers.

## Methods

### Research scenario

**Fig. 1:**
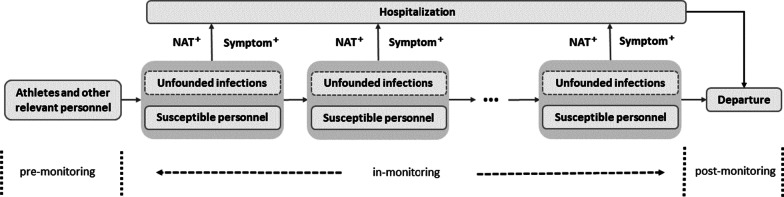
COVID-19 monitoring process of competition-related personnel during large-scale sports competitions

Based on the strict closed-loop management during the Tokyo 2020 Olympic Games and the upcoming Beijing 2022 Olympic and Paralympic Winter Games [8, 17], this study was designed for 18 surveillance scenarios for the competition-related personnel (including athletes, alternate athletes, and other team officials, acronym as CRP) from different countries as presented in Table 1. According to the Beijing 2022 Playbook Athletes and Officials [17], we assumed that the CRP would undergo unified health monitoring during the competition, including NAT monitoring (once a week to three times a day) and symptom monitoring (real-time dynamic monitoring including passive screening and self-report) [10] (Fig. 1). In this study, the frequency of NAT more than once in 2 days was defined as high frequency. In addition, to minimize the impact on non-virus-carrying athletes and ensure the smooth proceeding of the competition, we strengthen close-contact control by implementing an additional NAT within 14 days on the national sports delegation (NSD) with quarantined infections (Table 1).

**Table 1:** COVID-19 surveillance scenarios

Scenario	Frequency of NAT monitoring (the competition-related personnel)	Frequency of additional NAT monitoring (the national sports delegation with the quarantined infection)^a^
1	Every 7 days	–
2	Every 6 days	–
3	Every 5 days	–
4	Every 4 days	–
5	Every 3 days	–
6	Every 2 days	–
7	Once a day	–
8	Twice a day	–
9	Three times a day	–
10	Every 7 days	Once a day (Quarantine the infection)
11	Every 6 days	Once a day (Quarantine the infection)
12	Every 5 days	Once a day (Quarantine the infection)
13	Every 4 days	Once a day (Quarantine the infection)
14	Every 3 days	Once a day (Quarantine the infection)
15	Every 2 days	Once a day (Quarantine the infection)
16	Once a day	Once a day^b^ (Quarantine the infection)
17	Twice a day	Once a day (Quarantine the infection)
18	Three times a day	Once a day (Quarantine the infection)

^a^The competition-related personnel came from different national sports delegations. When an infection was found in the health monitoring, we would strengthen close-contact control, that was, we would increase the NAT frequency for people from the same national sports delegation for 14 days. ^b^When an infection was found, the infection was quarantined and the national sports delegation with the quarantined infection accepted NAT twice a day for 14 days. At the same time, other national sports delegations without the quarantined infection accepted NAT once a day

### Model assumptions

1. Individual NAT is negative before virus shedding.
2. The time required for NAT is ignored.
3. When individuals have specific clinical symptoms, they will be treated and isolated promptly.
4. Once infected with the SARS-CoV-2, the individual is immediately infectious.
5. Individuals all receive NAT at the customs and those with negative-test results are able to enter the model.
6. The individuals in the model will all receive NAT on the first day of monitoring. When leaving the model, a NAT report within 48 h is required.
7. The transmission rate between NSDs is two-thirds of that within NSDs.
8. The CRP are composed of 100 NSDs and enter the system in 60 groups. Each group has 100 people.

### Model description

**Fig. 2:**
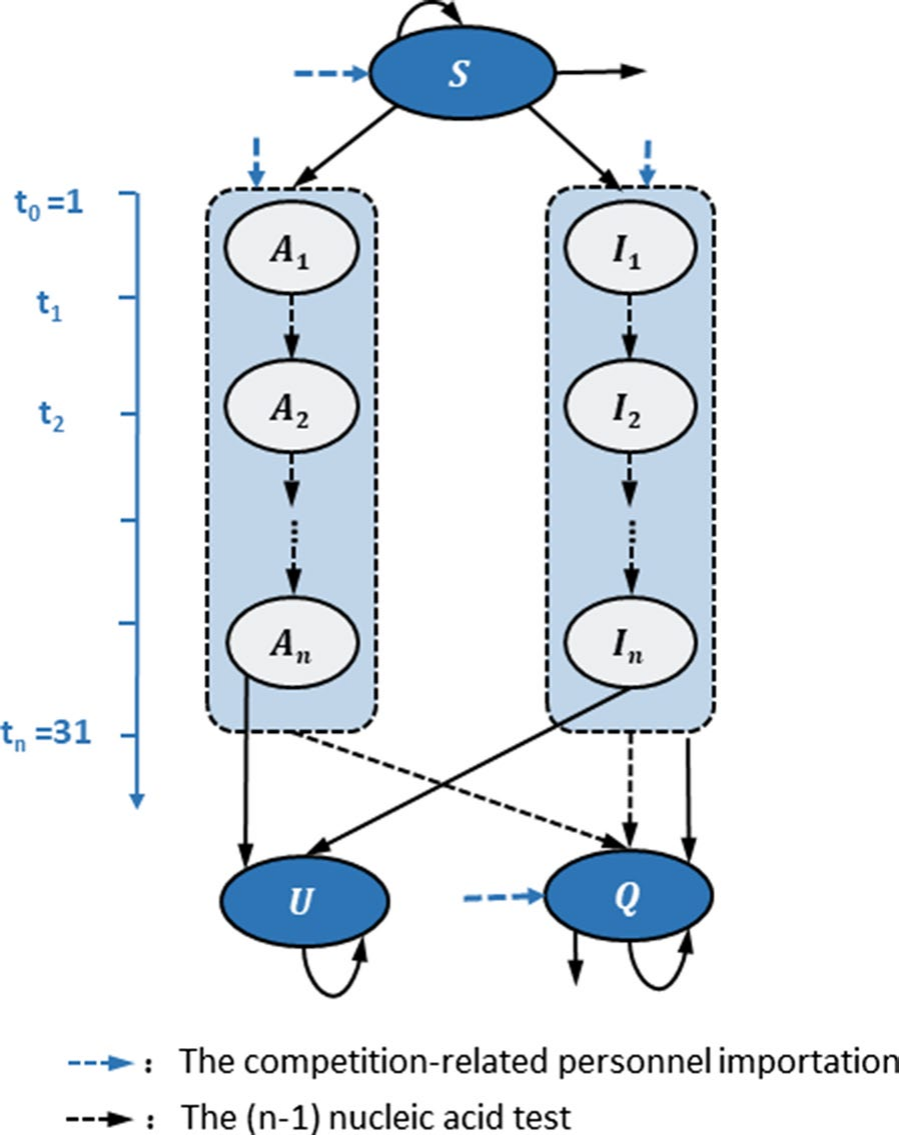
Stochastic dynamic model transfer chart of COVID-19 monitoring for competition-related personnel

Let *S, I, A, Q* denote the state of agents to be susceptible, to-be-symptomatic, asymptomatic and quarantined, respectively. Infections (*A* or *I*) were denoted by *U* as they left the system unfounded (Fig. 2)*.* Then the model evolves according to dynamic as follows (Additional file 1: Page 2–5).

1. Arrival: Arrivals consisting agents of state *I, A* and *S* would enter the system according to a fixed time schedule. As a result, the population of state *I, A* and *S* increased.
2. Departure: Agents left the system randomly after their sports events. Therefore, the population of state *I, A* and *S* decreased.
3. Transmission: A Poisson process was assigned to each infectious agent (*A* or *I*) to govern their transmission with rate *β*_*1*_*.* That is, the probability of occurrence for each infection’s transmission between time *t* and *t* + *δt* was given by.
  $$p(\delta t) = 1 - e^{{ - \beta_{1} \delta t}}$$
  Based on basic reproductive number *R*_*0*_*,* infectious period *IP,* vaccination rate *P*_*v*_ and vaccination efficacy against infection *P*_*i*_, *β*_*1*_ was equal to
  $$R_{0} /IP*\left( {1 - P_{v} } \right) \, + R_{0} /IP*P_{v} *\left( {1 - P_{i} } \right)$$
  (Additional file 1: page 3). As the transmission occurs, an individual that was not under quarantine would be selected. If the selected individual was *S*, he would be infected with probability 2/3 if the transmission occurred within NSD, 1 if the transmission occurred between NSDs. Considering the efficacy of vaccination against symptom *P*_*s*_ and asymptomatic rate of unvaccinated infectious agents *ρ*_1_, the state of infected S in our system would be changed to *A* with probability $\rho_{1} +(1-\rho_{1}) *P_{v} *P_{s} , {\text{with 1}}-(\rho_{1} +(1-\rho_{1}) *P_{v} *P_{s} )$ to *I* (Additional file 1: Figure S3).
4. Quarantine: The individual would be quarantined if he/she (1) Symptom onset: For each agent of State I who was exposed at time *T*, his/her pre-symptomatic incubation period was given by *T*_*I*_, which followed a Weibull distribution with scale parameter *λ* and shape parameter *k.* Once the system advanced at time *T*_*I*_ + *T,* the corresponding* I* would develop symptoms and became Q due to health monitoring. (2) Nucleic acid test (NAT): Once the agent of state *S* was infected, the virus in the agent would shed according to a Poisson process with rate *γ*. Thus, the time length of virus shedding followed exponential distributions with parameter *γ.* NAT applied after viral shedding would be able to detect the agent and move it into quarantine (Additional file 1: Figure S4).

Based on previous large-scale sports competitions, this study assumed that the surveillance strategy lasted for 31 days. In the first 10 days, we assumed that there were three groups of people going into the system every day. For the next 3 days, the number of groups increased to ten. I and A are randomly assigned to the arrival groups according to IIR. During the competition period (*14* ≤ *t* ≤ *29*), we assumed that there were five sports events every day. The individual who had finished the game at time *t*_1_ randomly chose a departure day randomly from day $\left\lceil {t_{1} } \right\rceil$ to the last day 31, and left at noon. See Additional file 1: Figure S5 for a detailed illustration.

### Outcome indicators

1. Accumulative infection (AI): The accumulative number of infections during the monitoring.
2. Accumulative unfounded number (AUN): The accumulative number of agents of state *U* during the health monitoring.
3. Accumulative detection ratio (ADR): The ratio of accumulative agents of state Q in accumulative infections.
  $$\mathrm{ADR}=\frac{AI-AUN}{AI}\times 100\%$$
4. Symptom detection ratio (SDR): The ratio of accumulative agents of state Q by symptom monitoring in accumulative detections.
5. Total cost (TC): It included the cost of health monitoring (HMC), the cost of medical treatment to the quarantined CRPs (MTC), the cost caused by the contact between the audience and the infectious CRPs (CC). The details of HMC, MTC and CC were in Additional file 1: page 6-7 [18–24].
6. Cost-effectiveness ratio (CER): It was used to determine compliance with the cost-effectiveness principle. The CER indicated the cost of each infection.
  $$\mathrm{CER}=\frac{TC}{AI}$$
7. Incremental cost-effectiveness ratio (ICER): Compared with Scenario 1 (NAT weekly without strengthening close-contact control) and Scenario 10 (NAT weekly and strengthening close-contact control) and corresponding Scenario 1–9 without strengthening close-contact control, it was the ratio of the reduction of total cost to the reduction of accumulative infections, and represented the cost savings of reducing an infection. Since the frequency of NAT monitoring every 7 days was commonly used [25], Scenario 1 and Scenario 10 were treated as control groups to explore whether increasing the frequency of NAT was cost-effective. And Scenario 1–9 were served as control groups to explore whether strengthening close-contact control was cost-effective
  $${\text{ICER}} = \frac{\Delta TC}{{\Delta AI}}.$$

### Statistical analysis

This study implemented the simulation of stochastic dynamic model, the sensitivity analysis, and subsequent data processing in MATLAB R2021a (Mathworks Inc., Natick, MA, USA) and Excel 2010 (Microsoft Corporation, Redmond, USA). Considering the uncertainty of different SARS-CoV-2 mutants, we used univariate sensitivity analysis including perturbations on initial infections rate (IIR), NAT accuracy ($\upmu$), infectious period (IP), basic reproductive number (${R}_{0}$), vaccination rate (${P}_{v}$), efficacy against infection (${P}_{i}$), efficacy against symptom (${P}_{s}$) and asymptomatic infection ratio (${\uprho }_{1}$). This sensitivity analysis focused on the change of the optimal scenario as parameters vary. The value range of parameters was shown in Table 3, and the details of IIR were shown in Additional file 1: Table S9 [26–37].

## Results

Based on the parameter values in Tables 2 and 3, this study simulated the proposed model for 1000 times and calculated the average and standard deviation (*SD*) of the outcome indicators in order to reduce randomness among different realizations (Additional file 1: Table S6, S7).

**Table 2:** Values and value range of initial cost parameters

Parameter	Value
Personnel salary (PS)	363.31 [18]
Nucleic acid test cost (NATC)	5.48 [19]
Number of audience (NA)	5000 [20–22]
Average medical cost (AMC)	2662.2 [23, 24]

The monetary unit of this study was United States Dollars (USD), USD 1 = RMB 6.3839, and 1 RMB ≈ USD 0.1566 on October 24, 2021. People infected by competition-related infections were mainly audience. Due to the short time horizon, discount rate was not considered

**Table 3:** Values and value ranges of model parameters

Parameter	Value	Value range
Initial infections rate (IIR)	0.020 [25]	0.001–0.020
$\lambda$	6.258 [26]	–
$k$	2.543 [26]	–
$\gamma$	2.9 [27–29]	–
$\mu$	0.92 [30]	0.69–1.00
${\rho }_{1}$	0.33 [31]	0.20–0.90
Infectious period (IP)	12.56 [26, 32]	6.00–20.00
Vaccination rate (${P}_{v}$)	0.58 [33]	0.20–1.00
Efficacy against infection (${P}_{i}$)	0.39 [34]	0.10–0.60
Efficacy against symptom (${P}_{s}$)	0.84 [35]	0.50–1.00
Basic reproductive number (${R}_{0}$)	3.38 [36]	2.54–12.00

### Trend of cases

**Fig. 3:**
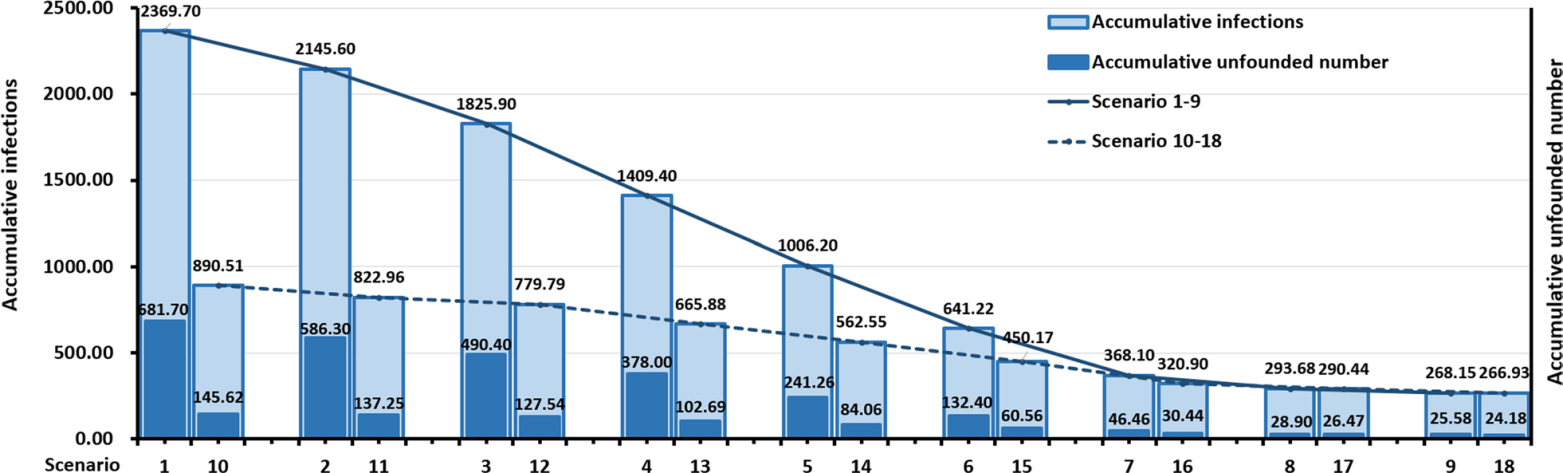
Composition of COVID-19 infections under different scenarios

Under Scenario 1‒9 and scenario 10‒18 in Fig. 3, with the increment in the NAT frequency, the AI and AUN were generally decreasing, and the decreasing degrees were gentler in Scenario 7‒9 and Scenario16‒18, respectively. Moreover, the downward trend of Scenario 1‒9 was more obvious than that of scenario 10‒18. In Scenario 1‒9, as the frequency of NAT increased, the AI decreased from 2369.70 to 268.15, and AUN decreased from 681.70 to 25.58. Meanwhile, in Scenario 10‒18, as the frequency of NAT increased, the AI decreased from 890.51 to 266.93 and AUN decreased from 145.62 to 24.18. In Table 4, the ADR of scenario 8–9 and 16–18 were all over 90% ranged from 90.16% to 90.94%. With the decrease in NAT frequency, the SDR significantly increased from 0.69% to 17.62%. In Scenario 1–4 (NAT weekly to once 4 days), the SDR was all above 10% with a range of 10.80% to 17.62%.

**Table 4:** COVID-19 transmission and cost effectiveness of different scenarios

Scenario	Initial infections	COVID-19 transmission				Cost effectiveness			
		Accumulative infection	Accumulative unfounded number	Accumulative detection ratio/%	Symptom detection ratio/%	Total cost (million dollars)	CER (million dollars)	ICER (million dollars)	
								Scenario 1–9^a^	Scenario 1, 10^b^
1	90	2369.70	681.70	71.23	17.62	133.94	0.0565	–	–
2	90	2145.60	586.30	72.67	16.48	115.33	0.0538	–	0.0830
3	90	1825.90	490.40	73.14	14.19	92.20	0.0505	–	0.0768
4	90	1409.40	378.00	73.18	10.80	65.43	0.0464	–	0.0713
5	90	1006.20	241.26	76.02	6.13	41.59	0.0413	–	0.0677
6	90	641.22	132.40	79.35	3.30	22.68	0.0354	–	0.0644
7	90	368.10	46.46	87.38	1.18	10.34	0.0281	–	0.0617
8	90	293.68	28.90	90.16	0.82	9.17	0.0312	–	0.0601
9	90	268.15	25.58	90.46	0.71	9.19	0.0343	–	0.0594
10	90	890.51	145.62	83.65	5.58	31.49	0.0354	0.0693	–
11	90	822.96	137.25	83.32	5.51	28.96	0.0352	0.0653	0.0375
12	90	779.79	127.54	83.64	5.20	27.31	0.0350	0.0620	0.0378
13	90	665.88	102.69	84.58	4.17	22.45	0.0337	0.0578	0.0403
14	90	562.55	84.06	85.06	2.80	18.15	0.0323	0.0528	0.0407
15	90	450.17	60.56	86.55	1.83	13.66	0.0303	0.0472	0.0405
16	90	320.90	30.44	90.51	0.99	8.92	0.0278	0.0301	0.0396
17	90	290.44	26.47	90.89	0.74	9.33	0.0321	−0.0476	0.0369
18	90	266.93	24.18	90.94	0.69	9.31	0.0349	−0.0924	0.0356

ICER, Incremental cost-effectiveness ratio; CER, Cost-effectiveness ratio

^a^These comparisons were Scenario 1–9 without strengthening close-contact control. ^b^These comparisons were Scenario 1 and Scenario 10 (once NAT weekly)

### Cost

**Fig. 4:**
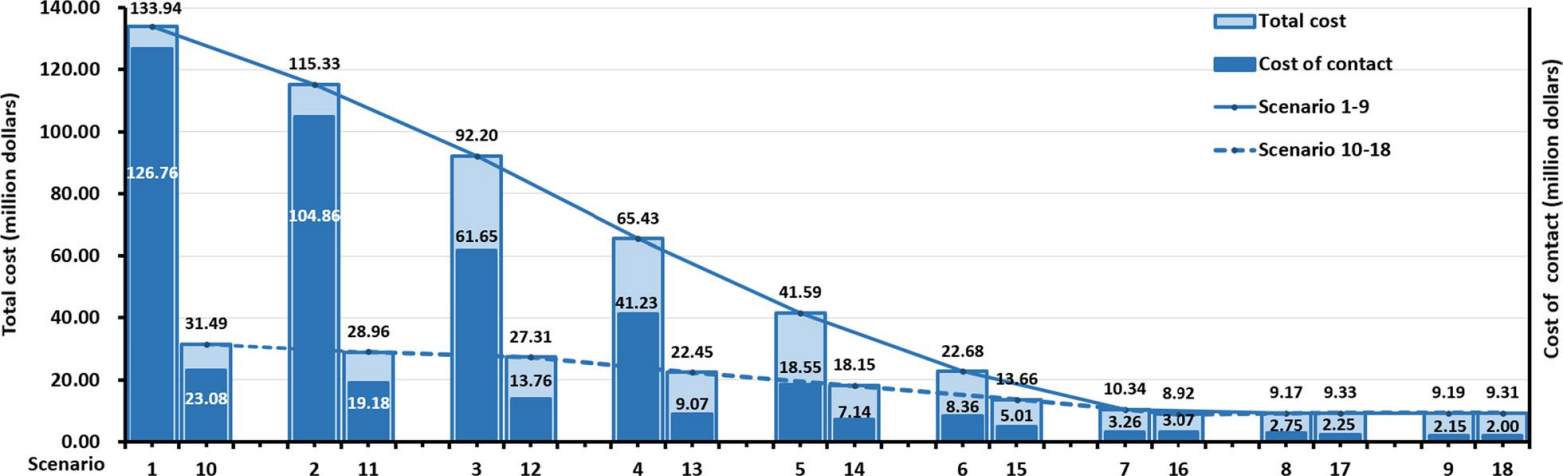
Composition of total cost under different scenarios

Under Scenario 1‒9 and Scenario 10‒18 in Fig. 4, with the increase of NAT frequency, the TC firstly decreased and then increased slightly, among which Scenario 8 and 16 were both in a relatively low position. In addition, the decline of TC in Scenario 1‒8 was greater than that of Scenario 10‒16. It is also worth noting that with the increase of NAT frequency, the proportion of the cost caused by AUN in TC decreased accordingly. In Table 4, TC reduced by USD 124 770 000 (93.15%) from Scenario 1 to Scenario 8, and the reduction on TC from Scenario 10 to Scenario 16 was USD 22 570 000 (71.67%). Scenario 16 had the lowest TC (USD 8 920 000), while Scenario 1 had the highest TC (USD 133 940 000) in 18 scenarios.

The CER of Scenario 1‒9 and 10‒18 both decreased first and then increased. The CER of Scenario 16 was the lowest of all scenarios. The cost to detect one infection was USD 27 800 in Scenario 16, while detecting out one infection cost USD 56 500 in Scenario 1 (Table 4). Compared with Scenario 1‒7, the ICER of the corresponding scenario with strengthening close-contact control (Scenario 10‒16) was greater than zero, and the incremental infections and the incremental costs of Scenario 10‒16 were less than zero, which meant Scenario 10–16 were more cost-effective than Scenario 17‒18. While the ICERs of Scenario 17‒18 were less than zero compared with scenario 8‒9. Compared with Scenario 10, the ICERs of Scenario 11–18 were all greater than zero, and the incremental infections and the incremental costs of Scenario 11‒18 were less than zero, with ranges of -67.55 to -623.58, and -2.35 to -22.18, respectively. Therefore, increasing NAT frequency was cost-effective (Table 4 and Additional file 1: Table S7).

### Sensitivity analysis

Sensitivity analysis showed that the optimal scenario was varied moderately according to the changes of ${R}_{0}$, IP, $\upmu$, ${P}_{v}$, and ${\uprho }_{1}$. When ${R}_{0}$ increased from 3 to 7, the optimal scenario changed from Scenario16 to Scenario 18. And accumulative infection ranged from 3851.92 to 4472.97 when ${R}_{0}$ were 7‒12. When $\upmu$ was 69%, ${\uprho }_{1}$ was 25%, IP was 9.42 and *P*_*v*_ was 44%, the optimal scenario became Scenario17. It was worth noting that when the IIR of CRP was low such as 0.1%, the corresponding optimal scenario would be adjusted to Scenario 15 (NAT for CRP twice a day, daily for the infected NSD). See Additional file 1: Table S8 for a detailed sensitivity analysis’ result.

## Discussion

This paper compared the cost-effectiveness of different surveillance scenarios using a stochastic dynamic model to optimize the surveillance strategies for successfully holding large-scale sports competitions. The results indicated that the most feasible and cost-effective scenario was NAT daily for CRP and an additional NAT daily for NSD accepting quarantined infections (Scenario 16) with an IIR less than 1.5%. Compared with Scenario 7 and Scenario 10, ICERs of Scenario 16 were both greater than zero, which meant that Scenario 16 could reduce AI and TC, and be more cost-effective due to implementing an additional NAT on the NSD with infections and increasing NAT frequency of CRP. Although AI of Scenario 8 and Scenario 9 was slightly lower than Scenario 16, Scenario 16 had a lower NAT frequency, which was more practical considering the competitive state of the athletes and their compliance with extremely high-frequency NAT. Meanwhile, the TC of Scenario 16 was the lowest among the 18 scenarios. Therefore, we proposed Scenario 16 as the most cost-effective scenario. Of course, if COVID-19 spread more severe during the competition, the host country should consider adjusting and strengthening the epidemic prevention measures in time, including increasing the frequency of NAT.

Sensitivity analysis indicated that vaccination was an effective approach to control the spread of the epidemic. A high vaccination rate will effectively reduce symptomatic infections as well as save the total cost. Although the Omicron variant has some immune escape ability [38], studies have shown that the vaccine remains fairly effective against (severe) symptoms [39]. In addition, China and many countries worldwide have started promoting the vaccination rate of COVID-19 vaccine booster shots to ensure high vaccine effectiveness.

In addition, the SARS-CoV-2 variant strains have different IIR, IP, ${\uprho }_{1}$, and ${R}_{0}$, which affect the selection of optimal scenario. At present, several globally circulating SARS-CoV-2 variant strains such as Omicron have emerged. Compared with the wild-type strain, Omicron is characterized by remarkably increased viral loads and transmissibility [40], which leads to more infections and costs. Therefore, implementing comprehensive non-pharmacological interventions including NAT at least once a day is an effective measure to ensure the successful hosting of sports competitions, such as the Tokyo 2020 Olympic Games, the National Basketball Association, and the Women’s National Basketball Association [41]. Specifically, the National Football League (NFL) successfully controlled the spread of COVID-19 by increasing the frequency of NAT (at least once per day) [42]. However, we could see that with low frequencies, two or three NATs every week, the Bundesliga professional football league (Bundesliga) and the Ultimate Fighting Championship (UFC) were still hailed as successful hostings of sporting events during the COVID-19, due to fewer athletes (1079 athletes in Bundesliga and 102 fighters in UFC) [43, 44], fewer gathering, and lower mobility. Unlike the Bundesliga and UFC, large-scale sports competitions face more complicated challenges in terms of gathering, mobility, and tougher epidemic prevention, in the case of which this study is more suitable. High-frequency NAT is vital for the timely case detection. However, such a high frequency of NAT is a significant challenge for the management and epidemic prevention personnel. Excellent organizational management systems, standardized sampling and testing operations, and sufficient budget are all key factors in accomplishing these tasks effectively. It is worth noting that when COVID-19 was severe, simply adding NAT could not effectively control the spread of the epidemic. It is necessary to consider adopting more stringent epidemic prevention measures, such as isolating close contacts, holding closed-loop competitions without audiences, and even reconsidering whether to hold competitions. As the number of the audience is set to be 0 in our system, the result shows that scenario 16 is still the most optimal choice.

This study had two innovations. First, this study assessed whether the comprehensive COVID-19 surveillance strategy with different NAT frequencies was cost-effective through a quantitative approach rather than based on previous medical experience. It helped determine the NAT frequency for large-scale sports competitions and provided a way to predict the epidemic spread under monitoring. Moreover, the model allowed the implementation of NATs at prefixed time points, which make it more realistic and practical compared with traditional stochastic models [15, 16], where the transitions from the infected to the quarantined followed the Poisson processes. The model we proposed here considered regular NAT scenarios for all the agents in the system. In this way, the agents in the system were detected at the same time rather than one by one according to Poisson processes, which was more reasonable in our real life.

## Study limitations

This study had the following limitations. Firstly, due to the continuous emergence of SARS-CoV-2 variants such as Omicron, initial infections rate, infectious period, basic reproduction number, and asymptomatic infection ratio might change, and even beyond the results of sensitivity analysis. Secondly, the application background of this model was under closed-loop management. If non-closed-loop management is implemented during large-scale competitions, then a model needs to be developed to depict both the competition-related and the community level transmissions, as well as the interactions between them. Third, the total cost of a COVID-19 surveillance strategy, including costs such as NAT sites, isolation rooms, and patient transfers, needs to be supplemented by more detailed research. Fourth, these surveillance strategies are specific to the CRP without regarding other personnel in the venues, such as broadcasters and the Olympic and Paralympic Family. In the future, more exploring can be focused on different variants, COVID-19 related cost, and non-closed-loop management.

## Conclusions

This is a pioneering study to evaluate the cost-effectiveness of COVID-19 surveillance strategies, especially high-frequency NAT. We observed that high-frequency NAT, such as bidaily, daily, and twice a day, is cost-effective. NAT daily for the CRP and strengthening close-contact control (an additional NAT daily for NSD with quarantined infections) could be prioritized as a variable in defense against COVID-19 at large-scale sports competitions. The prewarning COVID-19 surveillance strategy can help host countries detect virus carriers and prevent the spread of COVID-19.

## Supplementary Information

**Additional file 1: Table S1.** Description of the system variables. **Table S2.** Formal definition of the stochastic dynamic model. **Figure S3.** The diagram of transmission. **Figure S4.** The diagram of quarantine. **Figure S5.** The timeline of the system. **Table S6.** Standard deviation of COVID-19 transmission. **Table S7.** Standard deviation of cost effectiveness. **Table S8.** Univariate sensitivity analysis of different parameters. **Table S9.** Incidence rate of COVID-19 in different regions (December 2021).

## Data Availability

The datasets used and/or analysed during the current study are available from the corresponding author on reasonable request.

## Abbreviations

NAT: Nucleic acid test
CRP: Competition-related personnel
NSD: National sports delegation
USD: United States Dollar
AI: Accumulative infection
AUN: Accumulative unfounded number
ADR: Accumulative detection rate
SDR: Symptom detection rate
TC: Total cost
HMC: Cost of health monitoring
MTC: Cost of medical treatment to the quarantined CRPs
CC: Cost caused by the contact between the audience and the infectious CRPs
CER: Cost-effectiveness ratio
ICER: Incremental cost-effectiveness ratio
IIR: Initial infections rate
IP: Infectious period
*SD*: Standard deviation
NFL: The National Football League
Bundesliga: The Bundesliga professional football league
UFC: The Ultimate Fighting Championship
