# Supplementary material for: Cost-effectiveness analysis on COVID-19 surveillance strategy of large-scale sports competition
Source: Infect Dis Poverty. 2022 Mar 18;11:32. doi: 10.1186/s40249-022-00955-3 (PMC8931792; doi:10.1186/s40249-022-00955-3)
Supplement: Supplementary file 1 — Additional file 1: Table S1. Description of the system variables. Table S2. Formal definition of the stochastic dynamic model. Figure S3. The diagram of transmission. Figure S4. The diagram of quarantine. Figure S5. The timeline of the system. Table S6. Standard deviation of COVID-19 transmission. Table S7. Standard deviation of cost effectiveness. Table S8. Univariate sensitivity analysis of different parameters. Table S9. Incidence rate of COVID-19 in different regions (December 2021). [file 40249_2022_955_MOESM1_ESM.docx]

**Supplementary Materials**

[1. Model structure 2](#_Toc93050110)

[1.1 Description of the system variables 2](#_Toc93050111)

[1.2 Description of the stochastic dynamic model formal 2](#_Toc93050112)

[1.3 The diagram of transmission 4](#_Toc93050113)

[1.4 The diagram of quarantine 5](#_Toc93050114)

[1.5 The timeline of the system 5](#_Toc93050115)

[2. Description of outcome indicators 6](#_Toc93050116)

[2.1 Number of audiences 6](#_Toc93050117)

[2.2 Total cost 6](#_Toc93050118)

[3. Results in details 8](#_Toc93050119)

[3.1 Standard deviation of main result 8](#_Toc93050120)

[3.2 Univariate sensitivity analysis results 10](#_Toc93050121)

[3.3 Initial infection rate 12](#_Toc93050122)

# 1. Model structure

## 1.1 Description of the system variables

| Table S1. Description of the system variables | |
| --- | --- |
| Variable | Description |
| $S$ | Susceptible |
| $A_{n}$ | Asymptomatic infected persons waiting for the n^th^ test  (asymptomatic infected persons never show no symptoms) |
| $I_{n}$ | Symptomatic infected persons requiring the n^th^ test |
| $U$ | Unfounded  (Undetected infected population, false negative status) |
| $Q$ | Quarantined |

## 1.2 Description of the stochastic dynamic model formal

The population of the susceptible *S(t)*, to-be-symptomatic *I(t)*, asymptomatic *A(t),* quarantined *U(t)* and the unfounded infection *U(t)* at time *t=0* were all set to be *0*. We present the update of the system {*S(t), I(t), A(t), Q(t), U(t)*} below to formalize the description of the dynamic model.

Table S2. Formal definition of the stochastic dynamic model

| Symbol | Definition |
| --- | --- |
| *S(t)* | The population of the susceptible individuals |
| *I(t)* | The population of infections that will be symptomatic |
| *A(t)* | The population of asymptomatic infections |
| *Q(t)* | The population of the quarantined individuals |
| *U(t)* | The population of the infections that have left the system without quarantine |

1.2.1 Arrival

Based on the 2018 PyeongChang Winter Olympics, the study assumed a total of 6,000 the competition-related personnel (including athletes, alternate athletes, and other team officials, acronym as AO) from 100 countries. AO enter the Olympic Village in 60 batches to compete.

*[S, I, A, Q, U]* → *[S+100-I_1_-A_1_, I+I_1_, A+A_1_, Q, U]* every time a new group arrives according to the prefixed time schedule.

*I_1_～B(100, IIR*(1-*$\rho_{1}$*)*(1-P_v_*P_s_)), A_1_～B(100, IIR*(*$\rho_{1}$*+(1-*$\rho_{1}$*)*P_v_*P_s_))*

1.2.2 Departure

- *[S, I, A, Q, U]* → *[S-1, I, A, Q, U]* at noon on the day when an agent of state *S* choose to leave.
- *[S, I, A, Q, U]* → *[S, I-1, A, Q, U+1]* at noon on the day when an agent of state *I* choose to leave and *I* leaves the system with a negative NAT report within 48 hours.
- *[S, I, A, Q, U]* → *[S, I, A-1, Q, U+1]*at noon on the day when an agent of state *A* choose to leave and *A* leaves the system with a negative NAT report within 48 hours.
- *[S, I, A, Q, U]* → *[S, I, A-1, Q+1, U]*at noon on the day when an agent of state *A* choose to leave and *A* is tested out positive when receiving an extra *NAT* for departure.
- *[S, I, A, Q, U]* → *[S, I-1, A, Q+1, U]*at noon on the day when an agent of state *I* choose to leave and *I* is tested out positive when receiving an extra *NAT* for departure.

1.2.3 Transmission

- *[S, I, A, Q, U]* → *[S-1, I+1, A, Q, U]* at a Poisson rate

*(R_0_/IP*(1-P_v_ )+R_0_/IP*P_v_*(1-P_i_))*(I+A)*S/N*1/100*(1-*$\rho_{1}$*)*(1-P_v_*P_s_))*

*+(R_0_/IP*(1-P_v_) +R_0_/IP*P_v_*(1-P_i_) ) *(I+A)*S/N*99/100*2/3*(1-*$\rho_{1}$*)*(1-P_v_*P_s_))*

- *[S, I, A, Q, U]* → *[S-1, I, A+1, Q, U]* at a Poisson rate

*(R_0_/IP*(1-P_v_) +R_0_/IP*P_v_*(1-P_i_) )*(I+A)*S/N*1/100*(*$\rho_{1}$*+(1-*$\rho_{1}$*)*P_v_*P_s_)*

*+(R_0_/IP*(1-P_v_) +R_0_/IP*P_v_*(1-P_i_)) *(I+A) *S/N*99/100*2/3*(*$\rho_{1}$*+(1-*$\rho_{1}$*)*P_v_*P_s_)*

1.2.4 Quarantine

- *[S, I, A, Q, U]* → *[S, I-1, A, Q+1, U]* when an agent of state *I* develops symptom
- *[S, I, A, Q, U]* → *[S, I-1, A, Q+1, U]* when an agent of state *I* is detected out by NAT
- *[S, I, A, Q, U]* → *[S, I, A-1, Q+1, U]* when an agent of state *A* is detected out by NAT

## 1.3 The diagram of transmission

As the transmission rate was equal to the expected time length of an agent getting exposed by one infection, the transmission rate for each unvaccinated infection (*A* or *I*) was *R_0_/IP,* the quotient of basic reproductive number *R_0_* divided by infectious period *IP*. The transmission rate would be reduced to *(1-P_i_)R_0_/IP* for each vaccinated infection. Based on vaccination rate *P_v_* and efficacy of vaccination against infection *P_i_*, the universal transmission rate assigned to each infection in our system was equal to *R_0_/IP*(1-P_v_) + R_0_/IP*P_v_ *(1-P_i_)_._*

The Poisson process with rate *R_0_/IP*(1-P_v_) + R_0_/IP* P_v_ *(1-P_i_)* was assigned to each infection (*A* or *I*) to govern their transmission.

Similarity, the probability for an unvaccinated infected S changing his state to A was $\rho_{1}$, while the probability for a vaccinated infected S changing his state to A was $\rho_{1}$*+(1-*$\rho_{1}$*)*P_s._* Thus, for an infected S in our system, the probability for him changing his state to A was equal to

$\rho_{1}$*+(1-*$\rho_{1}$*)*P_v_*P_s._*


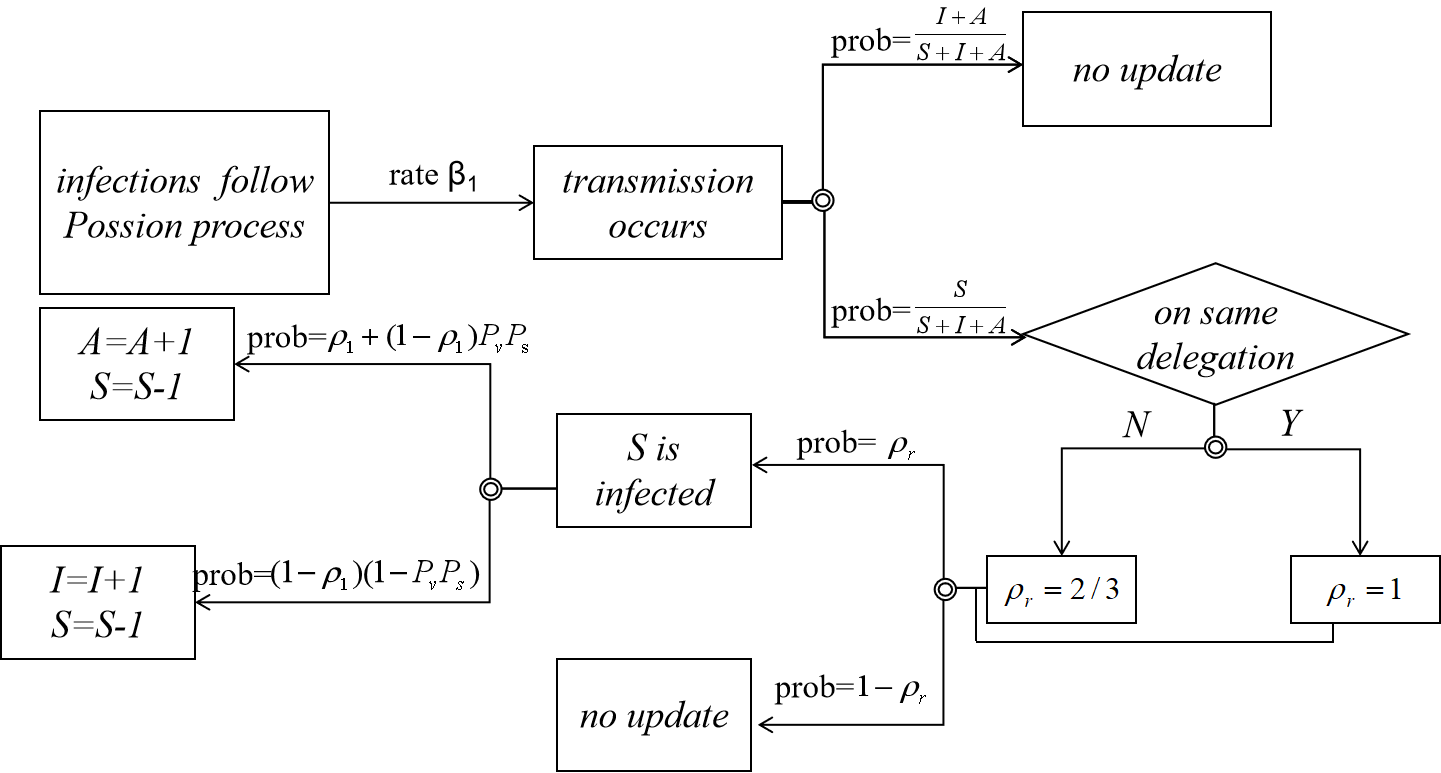
Figure S3. The diagram of transmission

##
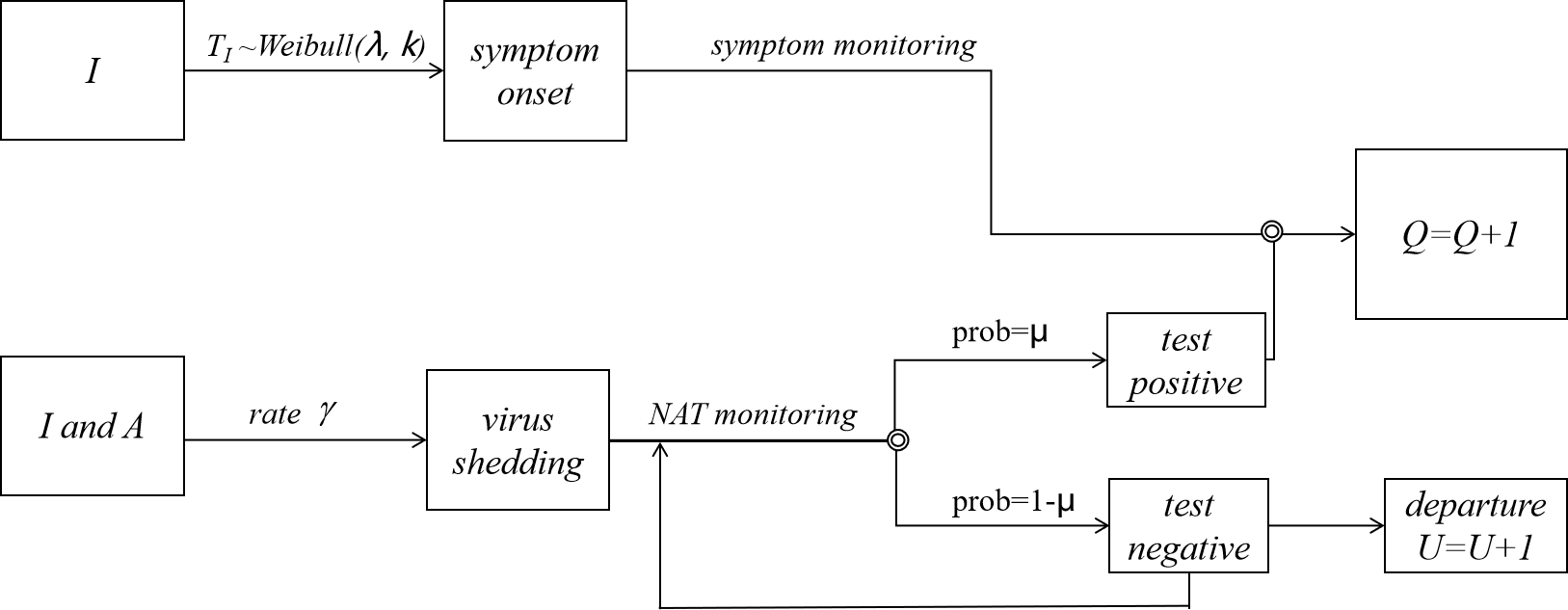
1.4 The diagram of quarantine

Figure S4. The diagram of quarantine

##
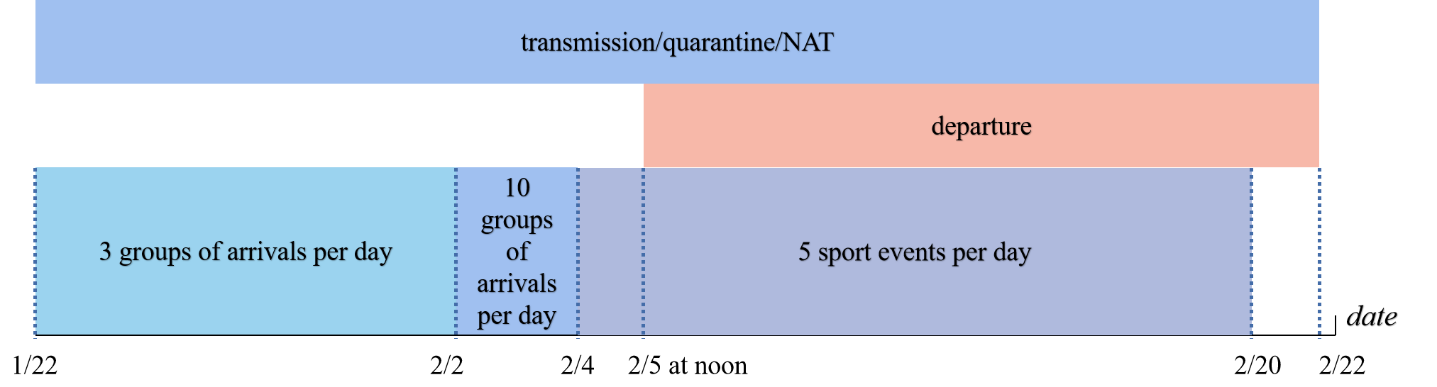
1.5 The timeline of the system

Figure S5. The timeline of the system

# 2. Description of outcome indicators

## 2.1 Number of audience

The Tokyo Organizing Committee had requested a maximum of 10,000 spectators per event ^[20]^. Total number of people in the Olympic Village (athletes, officials, etc.) in the 2021 Tokyo Olympics was 18000^[21]^. And total number of people in the Olympic Village (athletes, officials, etc.) in the 2018 PyeongChang Winter Olympics was 5900^[22]^. Based on the ratio of the total number of people in the Olympic Village of about 2:1, this study assumed that 5,000 people will be present at each event of the Beijing Winter Olympics.

## 2.2 Total cost

Total cost included the cost of health monitoring (HMC), the cost of medical treatment to the quarantined competition-related personnel (MTC), and the cost caused by the contact between the audience and the infectious competition-related personals (CC). HMC consisted of monitoring workers cost and NAT cost. The NAT cost came from aspects such as sample collecting, processing, and testing; information registration; treatment of medical waste; receiving clinical consultation ^[19]^. MTC was referred to all the expenses of treating the quarantined competition-related personals.

The cost caused by the contact between the audience and an infectious competition-related personnel (ACC) was referred to the NAT cost (NATC), personnel cost (PS), and average medical cost (AMC) caused by the spectators who were infected by an infectious CRP. AMC was referred to the expenses incurred in quarantine, isolation, treatment and testing from the time when an infected person was found to the time when he/she was discharged from hospital. The audience became infectious as he/she went into the competition venue and was infected by the competition-related personnel (CRP)’s infections who were not detected out in the system. Based on vaccination rate *P_v_* and efficacy of vaccination against infection *P_i_*, we pessimistically assumed that a CRP’s infection could infect $((1-P_{v}){\times R}_{0}+P_{v}\times(1-P_{i})\times R_{0})$ audiences. In actual competitions, due to short contact time between CRP and audience, long distance between CRP and audience, and wearing masks, we might have overestimated the number of audience’s infections. All the infected audiences had to be quarantined. At the same time, their close contacts would be required to undergo five regular NATs for the health monitoring according to Chinese regulations ^[24]^. Cost parameters and values were shown in Table 2.

$$ACC=PS\times\frac{NA}{50}+NATC\times5\times NA+((1-P_{v})\times R_{0}+P_{v}\times(1-P_{i})\times R_{0})\times AMC$$

# 3. Results in details

## 3.1 Standard deviation of main result

Table S6. Standard deviation of COVID-19 transmission

| Scenario | Initial infections | Accumulative infection | Standard deviation | Accumulative unfounded number | Standard deviation | Accumulative detection ratio/% | Standard deviation | Symptom detection ratio/% | Standard deviation |
| --- | --- | --- | --- | --- | --- | --- | --- | --- | --- |
| 1 | 90 | 2369.70 | 343.37 | 681.70 | 77.19 | 71.23 | 1.70 | 17.62 | 0.97 |
| 2 | 90 | 2145.60 | 330.02 | 586.30 | 74.72 | 72.67 | 1.61 | 16.48 | 0.97 |
| 3 | 90 | 1825.90 | 305.87 | 490.40 | 75.45 | 73.14 | 1.51 | 14.19 | 0.92 |
| 4 | 90 | 1409.40 | 281.86 | 378.00 | 78.03 | 73.18 | 1.76 | 10.80 | 1.00 |
| 5 | 90 | 1006.20 | 225.64 | 241.26 | 62.71 | 76.02 | 2.23 | 6.13 | 0.92 |
| 6 | 90 | 641.22 | 155.11 | 132.40 | 43.88 | 79.35 | 3.20 | 3.30 | 0.83 |
| 7 | 90 | 368.10 | 93.25 | 46.46 | 21.35 | 87.38 | 3.62 | 1.18 | 0.64 |
| 8 | 90 | 293.68 | 68.44 | 28.90 | 14.28 | 90.16 | 3.48 | 0.82 | 0.58 |
| 9 | 90 | 268.15 | 58.51 | 25.58 | 11.15 | 90.46 | 3.18 | 0.71 | 0.58 |
| 10 | 90 | 890.51 | 150.28 | 145.62 | 35.55 | 83.65 | 2.46 | 5.58 | 0.98 |
| 11 | 90 | 822.96 | 134.26 | 137.25 | 34.41 | 83.32 | 2.58 | 5.51 | 1.03 |
| 12 | 90 | 779.79 | 134.85 | 127.54 | 33.67 | 83.64 | 2.64 | 5.20 | 1.04 |
| 13 | 90 | 665.88 | 124.46 | 102.69 | 29.95 | 84.58 | 2.76 | 4.17 | 0.99 |
| 14 | 90 | 562.55 | 114.04 | 84.06 | 26.88 | 85.06 | 2.95 | 2.80 | 0.86 |
| 15 | 90 | 450.17 | 94.27 | 60.56 | 23.06 | 86.55 | 3.33 | 1.83 | 0.74 |
| 16 | 90 | 320.90 | 76.63 | 30.44 | 17.19 | 90.51 | 3.50 | 0.99 | 0.60 |
| 17 | 90 | 290.44 | 70.04 | 26.47 | 14.52 | 90.89 | 3.56 | 0.74 | 0.55 |
| 18 | 90 | 266.93 | 60.74 | 24.18 | 12.01 | 90.94 | 3.29 | 0.69 | 0.56 |

Table S7. Standard deviation of cost effectiveness

| Scenario | Initial infections | Total cost  (million dollars) | Standard deviation | CER  (million dollars) | Standard deviation | Incremental infections | | Incremental costs  (million dollars) | | ICER  (million dollars) | |
| --- | --- | --- | --- | --- | --- | --- | --- | --- | --- | --- | --- |
|  |  |  |  |  |  | Scenario 1-9* | Scenario 1, 10** | Scenario 1-9* | Scenario 1, 10** | Scenario 1-9* | Scenario 1, 10** |
| 1 | 90 | 133.94 | 20.99 | 0.0565 | 0.0027 | - | - | - |  | - | - |
| 2 | 90 | 115.33 | 18.79 | 0.0538 | 0.2699 | - | -224.10 | - | -18.61 | - | 0.0830 |
| 3 | 90 | 92.20 | 16.34 | 0.0505 | 0.2870 | - | -543.80 | - | -41.74 | - | 0.0768 |
| 4 | 90 | 65.43 | 13.66 | 0.0464 | 0.3405 | - | -960.30 | - | -68.50 | - | 0.0713 |
| 5 | 90 | 41.59 | 9.94 | 0.0413 | 0.3958 | - | -1363.50 | - | -92.35 | - | 0.0677 |
| 6 | 90 | 22.68 | 6.20 | 0.0354 | 0.4390 | - | -1728.48 | - | -111.25 | - | 0.0644 |
| 7 | 90 | 10.34 | 3.24 | 0.0281 | 0.4664 | - | -2001.60 | - | -123.60 | - | 0.0617 |
| 8 | 90 | 9.17 | 2.36 | 0.0312 | 0.4272 | - | -2076.02 | - | -124.76 | - | 0.0601 |
| 9 | 90 | 9.19 | 2.00 | 0.0343 | 0.4329 | - | -2101.55 | - | -124.74 | - | 0.0594 |
| 10 | 90 | 31.49 | 5.64 | 0.0354 | 0.3669 | -1479.19 | - | -102.45 |  | 0.0693 | - |
| 11 | 90 | 28.96 | 5.17 | 0.0352 | 0.3629 | -1322.64 | -67.55 | -86.37 | -2.53 | 0.0653 | 0.0375 |
| 12 | 90 | 27.31 | 5.01 | 0.0350 | 0.3685 | -1046.11 | -110.72 | -64.89 | -4.18 | 0.0620 | 0.0378 |
| 13 | 90 | 22.45 | 4.64 | 0.0337 | 0.4115 | -743.52 | -224.63 | -42.99 | -9.04 | 0.0578 | 0.0403 |
| 14 | 90 | 18.15 | 4.18 | 0.0323 | 0.4069 | -443.65 | -327.96 | -23.44 | -13.34 | 0.0528 | 0.0407 |
| 15 | 90 | 13.66 | 3.54 | 0.0303 | 0.4498 | -191.05 | -440.34 | -9.02 | -17.83 | 0.0472 | 0.0405 |
| 16 | 90 | 8.92 | 2.72 | 0.0278 | 0.4673 | -47.20 | -569.61 | -1.42 | -22.57 | 0.0301 | 0.0396 |
| 17 | 90 | 9.33 | 2.35 | 0.0321 | 0.4320 | -3.24 | -600.07 | 0.15 | -22.16 | -0.0476 | 0.0369 |
| 18 | 90 | 9.31 | 2.09 | 0.0349 | 0.4175 | -1.22 | -623.58 | 0.11 | -22.18 | -0.0924 | 0.0356 |

NOTE: * These comparisons were Scenario 1-9 without strengthening close-contact control. ** These comparisons were Scenario 1 and Scenario 10 (once NAT weekly).

## 3.2 Univariate sensitivity analysis results

Table S8. Univariate sensitivity analysis of different parameters

| Parameter | Value | Optimal scenario | Accumulative infection | Accumulative unfounded number | Accumulative detection ratio/% | Symptom detection ratio/% | Total cost  (million dollars) | CER  (million dollars) |
| --- | --- | --- | --- | --- | --- | --- | --- | --- |
| Initial infectious rate | 0.10 | 15 | 40.05 | 7.75 | 80.66 | 2.79 | 1.74 | 0.0435 |
|  | 0.30 | 16 | 70.88 | 8.08 | 88.60 | 1.05 | 2.55 | 0.0360 |
|  | 0.50 | 16 | 120.62 | 13.94 | 88.44 | 1.06 | 3.93 | 0.0326 |
|  | 0.70 | 16 | 159.52 | 18.09 | 88.66 | 1.02 | 4.92 | 0.0308 |
|  | 1.00 | 16 | 226.80 | 24.29 | 24.73 | 3.62 | 6.09 | 0.0269 |
|  | 1.50 | 16 | 320.90 | 30.44 | 90.51 | 0.99 | 8.92 | 0.0278 |
|  | 2.00 | 16 | 429.90 | 41.07 | 90.45 | 0.97 | 11.12 | 0.0259 |
| NAT accuracy | 0.69 | 17 | 304.07 | 30.90 | 89.84 | 0.95 | 11.78 | 0.0289 |
|  | 0.80 | 17 | 295.39 | 29.07 | 90.16 | 0.92 | 11.00 | 0.0278 |
|  | 0.90 | 16 | 328.86 | 33.22 | 89.90 | 1.04 | 11.67 | 0.0265 |
|  | 0.92 | 16 | 320.90 | 30.44 | 90.51 | 0.99 | 8.92 | 0.0278 |
|  | 1.00 | 16 | 302.27 | 27.98 | 90.74 | 0.87 | 10.30 | 0.0254 |
| Asymptomatic infection ratio | 0.20 | 17 | 271.97 | 24.14 | 91.12 | 0.94 | 9.72 | 0.0267 |
|  | 0.25 | 17 | 278.87 | 26.07 | 90.65 | 0.89 | 10.07 | 0.0269 |
|  | 0.33 | 16 | 320.90 | 30.66 | 90.45 | 0.97 | 10.96 | 0.0255 |
|  | 0.40 | 16 | 312.76 | 30.76 | 90.16 | 0.82 | 10.97 | 0.0262 |
|  | 0.41 | 16 | 314.29 | 31.87 | 89.86 | 0.81 | 11.19 | 0.0266 |
|  | 0.60 | 16 | 315.28 | 31.76 | 89.93 | 0.59 | 11.31 | 0.0268 |
|  | 0.80 | 16 | 322.09 | 33.25 | 89.68 | 0.27 | 11.46 | 0.0265 |
|  | 0.90 | 16 | 322.85 | 32.87 | 89.82 | 0.13 | 11.49 | 0.0266 |
| Infectious period (IP) | 6.00 | 18 | 3896.33 | 572.98 | 85.29 | 0.38 | 179.62 | 0.0344 |
|  | 8.00 | 18 | 1431.99 | 330.08 | 76.95 | 0.39 | 61.38 | 0.0320 |
|  | 9.00 | 17 | 951.80 | 210.93 | 77.84 | 0.50 | 40.76 | 0.0320 |
|  | 9.42 | 17 | 746.11 | 152.47 | 79.56 | 0.57 | 31.43 | 0.0314 |
|  | 10.00 | 16 | 702.11 | 128.35 | 81.72 | 0.72 | 28.65 | 0.0305 |
|  | 12.56 | 16 | 320.90 | 30.44 | 90.51 | 0.99 | 8.92 | 0.0278 |
|  | 14.00 | 16 | 253.47 | 19.60 | 92.27 | 1.04 | 8.44 | 0.0248 |
|  | 15.70 | 16 | 193.63 | 10.49 | 94.58 | 1.17 | 6.51 | 0.0251 |
|  | 18.00 | 16 | 172.01 | 7.10 | 95.87 | 1.22 | 4.92 | 0.0213 |
|  | 20.00 | 15 | 181.18 | 7.57 | 95.82 | 2.56 | 5.47 | 0.0225 |
| Vaccination rate | 0.20 | 18 | 409.72 | 52.50 | 87.19 | 0.97 | 16.08 | 0.0293 |
|  | 0.40 | 17 | 344.08 | 34.23 | 90.05 | 0.93 | 12.93 | 0.0281 |
|  | 0.44 | 17 | 326.34 | 36.00 | 88.97 | 0.99 | 12.07 | 0.0276 |
|  | 0.58 | 16 | 320.90 | 30.44 | 90.51 | 0.99 | 8.92 | 0.0278 |
|  | 0.60 | 16 | 306.84 | 29.22 | 90.48 | 0.89 | 10.71 | 0.0260 |
|  | 0.73 | 16 | 277.50 | 23.78 | 91.43 | 0.77 | 9.30 | 0.0250 |
|  | 0.80 | 16 | 248.15 | 18.35 | 92.61 | 0.68 | 7.88 | 0.0237 |
|  | 1.00 | 16 | 205.03 | 11.75 | 94.27 | 0.35 | 6.01 | 0.0219 |
| Efficacy against infection | 0.10 | 18 | 440.74 | 58.25 | 86.78 | 0.59 | 17.32 | 0.0293 |
|  | 0.20 | 17 | 398.96 | 55.64 | 86.05 | 0.72 | 15.45 | 0.0289 |
|  | 0.29 | 16 | 385.48 | 46.21 | 88.01 | 0.93 | 14.05 | 0.0272 |
|  | 0.39 | 16 | 320.90 | 30.44 | 90.51 | 0.99 | 8.92 | 0.0278 |
|  | 0.49 | 16 | 259.33 | 19.79 | 92.37 | 1.11 | 8.56 | 0.0246 |
|  | 0.60 | 16 | 224.67 | 14.46 | 93.56 | 1.17 | 7.17 | 0.0238 |
| Efficacy against symptom | 0.50 | 16 | 313.14 | 31.10 | 90.07 | 1.36 | 11.30 | 0.0269 |
|  | 0.60 | 16 | 313.88 | 30.14 | 90.40 | 1.27 | 10.97 | 0.0261 |
|  | 0.63 | 16 | 314.74 | 28.74 | 90.87 | 1.17 | 10.94 | 0.0260 |
|  | 0.70 | 16 | 315.59 | 27.33 | 91.34 | 1.14 | 10.92 | 0.0258 |
|  | 0.80 | 16 | 317.29 | 24.86 | 92.16 | 1.03 | 10.87 | 0.0256 |
|  | 0.84 | 16 | 320.90 | 30.44 | 90.51 | 0.99 | 8.92 | 0.0278 |
|  | 0.90 | 16 | 324.80 | 30.44 | 90.63 | 0.91 | 11.07 | 0.0254 |
|  | 1.00 | 16 | 321.21 | 32.91 | 89.75 | 0.78 | 10.92 | 0.0254 |
| Basic reproductive number | 2.54 | 16 | 185.78 | 8.65 | 95.34 | 1.20 | 5.38 | 0.0216 |
|  | 3.00 | 16 | 250.99 | 18.53 | 92.62 | 1.10 | 8.17 | 0.0243 |
|  | 3.38 | 16 | 320.90 | 30.44 | 90.51 | 0.99 | 8.92 | 0.0278 |
|  | 4.19 | 17 | 532.94 | 89.19 | 83.27 | 0.65 | 21.89 | 0.0307 |
|  | 5.00 | 17 | 1264.86 | 307.39 | 75.70 | 0.49 | 56.13 | 0.0331 |
|  | 7.00 | 18 | 3851.92 | 586.11 | 84.78 | 0.37 | 184.14 | 0.0357 |
|  | 10.00 | 18 | 4454.08 | 165.64 | 96.28 | 0.75 | 173.04 | 0.0290 |
|  | 12.00 | 18 | 4472.97 | 103.68 | 97.68 | 0.83 | 145.79 | 0.0243 |

## 3.3 Initial infection rate

In this study, we assumed that the initial infection rate (IIR) is assumed to equal the undetected COVID-19 infection rate. We pessimistically took 1.5% as the main result ^[25]^.

Table S9. Incidence rate of COVID-19 in different regions (December 2021)

|  | Incidence rate/% |
| --- | --- |
| Africa | 0.06 |
| Asia | 0.05 |
| Europe | 1.50 |
| North America | 0.78 |
| Oceania | 0.26 |
| South America | 0.13 |
| World | 0.25 |
